# ALA Nanoemulsion Prepared By Microfluidic for Ocular Delivery: *In-Vitro* and *In-Vivo* Studies

**DOI:** 10.34172/apb.46273

**Published:** 2026-05-09

**Authors:** Javad Behmadi, Amiremad Kheirieh, Asma Mahmoudi, Mojtaba Abrishami, Seyed Ali Mousavi Shaegh, Amirhossein Abbasi, Bizhan Malaekeh-Nikouei

**Affiliations:** ^1^Student Research Committee, School of Pharmacy, Mashhad University of Medical Sciences, Mashhad, Iran; ^2^Department of Pharmaceutics, School of Pharmacy, Mashhad University of Medical Sciences, Mashhad, Iran; ^3^Nanotechnology Research Center, Pharmaceutical Technology Institute, Mashhad University of Medical Sciences, Mashhad, Iran; ^4^Eye Research Center, Mashhad University of Medical Sciences, Mashhad, Iran; ^5^Laboratory of Microfluidics and Medical Microsystems, BuAli Research Institute, Mashhad University of Medical Sciences, Mashhad, Iran; ^6^Orthopedic Research Center, Ghaem Hospital, School of Medicine, Mashhad University of Medical Sciences, Mashhad, Iran

**Keywords:** Alpha-lipoic acid, Cationic nano-emulsion, Microfluidic, Ophthalmic drug delivery, Ocular diseases

## Abstract

**Purpose:**

Different ocular diseases, such as diabetic retinopathy and cataracts, could be induced by oxidative stress. Alpha-lipoic acid (ALA) exhibits antioxidant activity; however, its low aqueous solubility limits its ophthalmic use. Cationic nano-emulsions (NEs) have emerged as an effective method to enhance the bioavailability of drugs, particularly those with low water solubility. Traditional methods for synthesizing nano-emulsions typically involve multiple steps. In contrast, deployment of the microfluidic technique enables nanoassembly of NEs in a single step through a micromixer with improved efficiency and minimized reagents.

**Methods:**

In this research, ALA cationic NEs were synthesized utilizing a microfluidic device, with variations in total flow rates. The resulting formulations were characterized based on their size. The formulation identified as the most optimal underwent further assessment regarding its physicochemical properties. Following accelerated stability assessments, animal studies were performed to evaluate the safety of the formulation.

**Results:**

The findings indicated a loading efficiency of 79.92% for the optimized formulation. The synthesized nanoparticles exhibited satisfactory characteristics in terms of pH, refractive index, osmolality, and rheological properties. Ex vivo release test revealed the formulation released up to 59.3% of ALA. Furthermore, ALA was detected in both vitreous and aqueous humor during in vivo experiments. The formulation successfully passed accelerated stability tests and demonstrated good tolerance in the ocular environment of rabbits throughout the animal study.

**Conclusion:**

The cationic ALA nanoemulsion synthesized by microfluidics demonstrates favorable characteristics, along with high stability and tolerability, which have proven beneficial for subsequent evaluations.

## Introduction

 The human eye is particularly susceptible to oxidative stress. Several ocular diseases, including age-related macular degeneration, diabetic retinopathy, and cataracts, are associated with oxidative stress.^1^ Various risk factors, including inflammation, sunlight exposure, aging, genetics, and environmental pollution, contribute to oxidative stress.^2^ It has been shown that oral antioxidants can decrease the incidence of these diseases or alleviate their symptoms.^3^

 Alpha-lipoic acid (ALA), also known as thioctic acid, is a natural organosulfur compound known for its numerous beneficial properties, one of which is its potent antioxidant capacity in both lipophilic and hydrophilic environments.^4,5^ Through the formation of mixed disulfides, ALA can act as a redox couple, facilitating alterations in protein conformations. Additionally, ALA exhibits the ability to scavenge free radicals, chelate metal ions, inhibit lipid peroxidation, and restore levels of glutathione. Also, ALA improves the management of glucose and ascorbate, boosts endothelial nitric oxide synthase activity, and initiates Phase II detoxification. On the other hand, ALA reduces the expression of vascular cell adhesion molecule 1 (VCAM-1).^6^ According to this mechanism, ALA is currently administered orally for the management of diabetic polyneuropathy and various related complications.^7^ However, studies have shown that the therapeutic efficacy of ALA is restricted due to limitations in its pharmacokinetic profile. Research indicates that ALA possesses a brief half-life and a bioavailability of roughly 30% when taken orally. This is attributed to factors such as hepatic degradation, diminished solubility of ALA, and instability within the gastrointestinal tract.^4^ On the other hand, developing an ophthalmic formulation of ALA poses challenges owing to its low solubility in water, instability during light exposure, and reactivity with other components. ALA has low ocular bioavailability due to eye defense mechanisms and a low drug concentration gradient.^8^

 Nanotechnology is regarded as a promising strategy for addressing these challenges through enhancing solubility and, consequently, therapeutic efficacy.^9,10^ Cationic nano-emulsions could present a particularly effective method for improving the bioavailability of ocular drugs. To this end, the precorneal residence time of eye drop nanoformulations is prolonged through electrostatic interactions between the positively charged oil nano-droplets and the negatively charged epithelial surface of the eye. However, the number of nanotechnology-based products that have successfully entered the market remains limited, primarily because of the existing complexities involved in the scale-up production pipeline, which requires multiple manufacturing steps and equipment.^11,12^

 Microfluidics has been emerged as a revolutionary technology for the production of nanoparticles with diverse shapes, sizes, and morphologies.^13^ Microfluidic technologies encompass systems designed to process or manipulate fluids through microchannels.^14^ This innovative approach streamlines the fabrication of nanoparticles by eliminating the need to employ multiple devices and size-reduction equipment throughout the nanoassembly process.^15,16^ It allows for controlled mixing of formulation solutions in a micromixer to control size and PDI by manipulating flow rates of the solutions; thus, batch-to-batch variations decrease dramatically, and the overall production efficiency increases.^13^

 The current research focused on the development of an efficient ophthalmic ALA cationic nano-emulsion utilizing a microfluidic method. The resulting nanoparticles were characterized and assessed for their physicochemical properties. Additionally, the formulation’s permeability through bovine cornea was examined in a laboratory setting. Ultimately, the safety of the final product was evaluated in rabbits to confirm the planning of subsequent clinical studies.

## Materials and Methods

###  Materials

 ALA, Polysorbate 80 (Tween 80), and Cetalkonium chloride (CTK) were obtained from Sigma Aldrich (Germany). Span 80, medium-chain triglyceride (MCT) oil, vitamin E, and glycerin were sourced from Pars Sadra (Iran). HPLC-grade acetonitrile was procured from DAEJUNG Company Ltd (Korea), while methanol was supplied by Scharlau Chemie S.A. (Spain). Chloroform and ammonium acetate were acquired from Merck Company (Germany). Male New Zealand white rabbits were provided by the Razi Vaccine and Serum Research Institute (Mashhad, Iran) and were maintained under standard housing conditions. All other chemicals used in this study were of analytical grade.

###  Preparation of ALA Cationic Nano-Emulsion 

 ALA cationic nano-emulsions were prepared using a microfluidic approach. To start with, formulation solutions, including aqueous and oil phases, were prepared. The components of the aqueous and oil phases of the formulation are described in Table 1. To prepare the oil phase, MCT oil and CTK were mixed. Then, alpha-lipoic acid was weighed and dissolved in ethanol, resulting in a clear yellow solution. This yellow solution was added to the mixture of MCT and CTK. Following this, vitamin E, Span 80, and Tween 80 were incorporated into the oil phase, and the mixture was vortexed until it achieved a completely clear and particle-free consistency. To prepare the aqueous phase, glycerin was added to a Falcon tube and diluted with deionized water to achieve the desired volume. The oil and water phases were heated to 65 °C using a water bath and subsequently transferred into appropriate syringes. Filled solutions were connected to a micromixing cartridge with two inlets and one outlet. Then, the cartridge with two connected syringes was inserted into the Nanosynthesizer apparatus (INSIGHT, NanoSynthes Co.), as it enabled precise injection of formulation solutions into the mixing cartridge. Rapid mixing of aqueous and oil phases occurred within the cartridge, and nano-emulsions with encapsulated ALA were prepared and collected at the outlet of the cartridge, since it accommodated a micromixer (Figure S1). The Nanosynthesizer apparatus allowed for heating the two syringes and the cartridge during the injection process at a desired temperature of 65 °C to maintain the quality of nanoemulsions. A temperature drop in formulation solutions may deteriorate the uniformity of the nanoparticles. Injection of the solutions into the mixing cartridge was performed at a ratio of 1:9 (oil: water), and the first few seconds of the injection process were separated as waste first seconds were discarded to improve PDI of the collected sample. To find an optimum flow rate of the two solutions to produce nano-emulsions, a range of flow rates was examined, including 10, 12, 14, 16, and 18 ml/min. Finally, the total flow rate (TFR) of 18 was employed for nanoparticle preparation. It is worth noting that the size and PDI of prepared particles using the microfluidic method, i.e., rapid micromixing, are dependent on the flow rate ratio (FRR), the TFR of the solutions mixed in the cartridge, the temperature of the solutions, as well as the composition of the formulation solutions. The temperature of the solutions was maintained at 65 °C for all procedures. pH of resulting nanoemulsions was adjusted to 6.5 ± 0.1 using 0.1 N NaOH, and they were stored under inert gas.

**Table 1:** Composition of aqueous and oil phases of ALA cationic nanoemulsion.

**Oil phase**	**Quantity**
Alpha-lipoic acid (ALA)	200 mg
Cetalkonium chloride (CTK)	20 mg
Medium-chain triglycerides (MCT oil)	1 ml
Ethanol	300 μl
Span 80	390 μl
Tween 80	390 μl
Vitamine E	0.1 % w/v
**Water phase**	**Quantity**
Glycerin	500 μl
D.W.	Up to 10 ml

###  Size and Zeta Potential

 The analysis of particle size was conducted using the dynamic light scattering (DLS) technique, along with the assessment of zeta potential for the nanoparticles, utilizing Zetasizer Nano ZS (UK). Samples were prepared by adding 10 µL of the cationic nanoemulsion formulation to 990 µL of deionized water, followed by bath sonication for 5 minutes. For the measurement of zeta potential, we used deionized water with a pH of 6.2 during the measurements. Additionally, the number of runs was 12. In the DLS protocol employed in this study, the dispersant’s refractive index was 1.330, and the viscosity was assumed to be 0.8872 cP, with scattering angles of 173 °C and 90 °C. The cuvette’s external dimensions were 12.5 × 12.5 mm, and its internal dimensions were 10 × 10 mm, resulting in a path length of 10 mm. Dilution media were classified based on their dielectric constant: polar dispersants, with a dielectric constant greater than 20 (such as ethanol and water), and non-polar or low-polarity dispersants, with a dielectric constant less than 20 (such as hydrocarbons and higher alcohols). In this study, deionized water, a polar aqueous medium, was used. The purpose of sample preparation was to preserve the original surface condition during analysis, which involved using low sample concentrations. Under these conditions, the viscosity of the dispersant can be considered equal to that of the sample. This assumption generally holds true at high dilution levels; however, for sample concentrations exceeding 0.1%, the actual viscosity of the sample should be measured and applied.

###  FESEM Electron Microscopy

 Field emission scanning electron microscopy (FESEM) was employed to analyze and visualize the nanoparticles present in the formulation. This technique utilizes electrons emitted from a source that collide with coated nanoparticles in a vacuum under a high electrical field gradient. The formulations were diluted at ratios of 1:10 and 1:100 with filtered deionized water. The resulting solutions were then deposited onto aluminum foil and allowed to air dry. To enhance contrast and clarity, dried nanoparticles were coated with gold particles and subsequently evaluated using FESEM.^17^

###  Determination of Incorporated ALA

 For the quantification of incorporated ALA, a SHIMADZU DGU-20A5R high-performance liquid chromatography (HPLC) system (JPN) was employed in conjunction with a 4.6 × 250 mm^2^ reverse-phase stainless steel C18 column. The mobile phase consisted of a 50:50 (v/v) mixture of acetonitrile and 20 mM ammonium acetate at pH 4.6, with a flow rate maintained at 0.8 ml/min at ambient temperature. Standard concentration series were established using 125, 250, 500, 750, and 1000 µg/ml of ALA, prepared in a 1:1 mixture of acetonitrile and 20 mM ammonium acetate, from which a standard curve was generated. Samples were appropriately diluted in a methanol: chloroform (1:1) solution to ensure clarity, with each injection volume set at 20 µl.

###  Physicochemical Characterization of Cationic Nano-Emulsions

####  Evaluation of Rheological Properties

 Rheological assessments were conducted using a Brookfield Rotation Stairs Rheometer (GER) at a temperature of 34 ± 0.5 °C. A continuous shear rate (γ) variation was applied within the range of 0 to 900 s⁻¹ throughout 10 cycles, each lasting 1 second, during which the resultant shear stress (σ) was recorded. Subsequently, the viscosity (η) of the cationic nanoemulsion preparation, which exhibited Newtonian flow characteristics, was determined using the following equation: η = σ/γ.^18^

####  pH Measurements

 A pH meter model MI 151 from MARTINI Instruments (USA) was used to determine the pH of the synthesized nanoemulsion.

####  Refractive Index Measurements

 The determination of the refractive index was conducted using the BPTR 100 PrismaTech (Iran) at a controlled temperature of 25 °C.

####  Osmolality Determination

 Micro Osmometer (K-7400S, KNAUER, GER) is used to determine the osmolality of the synthesized nanoemulsion.

####  Ex Vivo Release Test

 To investigate the transition of the formulation from the bovine cornea, an experimental setup was designed and fabricated (Figure S2). The initial part of the setup includes a pump. 5,000 µl of balanced salt buffer is filled in a 5 ml syringe and inserted into the chamber of the pump. The pump operates at a flow rate of 2 µl/min over 24 hours. This device was designed and fabricated to model *in vitro* ocular flow. The design process prioritized ease of fabrication and usability. Initially, the device components were modeled using CATIA software, which facilitated the elimination of specialized machining tools and minimized the operations required for part fabrication. Subsequently, CNC machining was employed to manufacture the various components of the device. As illustrated in Figure 1, the device comprises three distinct components interconnected by screws. Part A represents the corneal surface, where 500 µl of the cationic NE is applied, Part B corresponds to the anterior cavity, and Part C pertains to the posterior cavity. Two membranes strategically separate these components: The bovine cornea was located between Part A and Part B, while the dialysis bag was inserted between Part B and Part C. This design enables researchers to investigate the transport of a drug through the cornea, anterior cavity, and posterior cavity in an *in vitro* setting. The dimensions of the components were selected to accurately reflect the dimensions of the counterparts of the human eye. Specifically, the posterior and anterior cavities have volumes of 4.2 ml and 0.2 ml, respectively.^19^ Furthermore, the device is designed to simulate the natural physiological flow in the eye. To facilitate this, two ports serve as an inlet and outlet, located in Parts B and C, respectively, and connected with 1.5 mm outer diameter tubing. Effective sealing is crucial due to fluid flow within the device. Therefore, the device has been designed to be fully sealed using fitted rubber washers. Polymethyl methacrylate (PMMA) was chosen for the fabrication of this device owing to its properties. PMMA has a high transparency of 92% for visible light, making it suitable for making devices that require continuous interior observation and monitoring. PMMA is a thermoplastic in which its mechanical/structural properties allow for conducting high-precision machining and laser ablation cutting in research labs and industrial settings. Importantly, it has high biocompatibility, making it suitable for *in vitro* studies and cell cultures. ^20,21^

**Figure 1:**
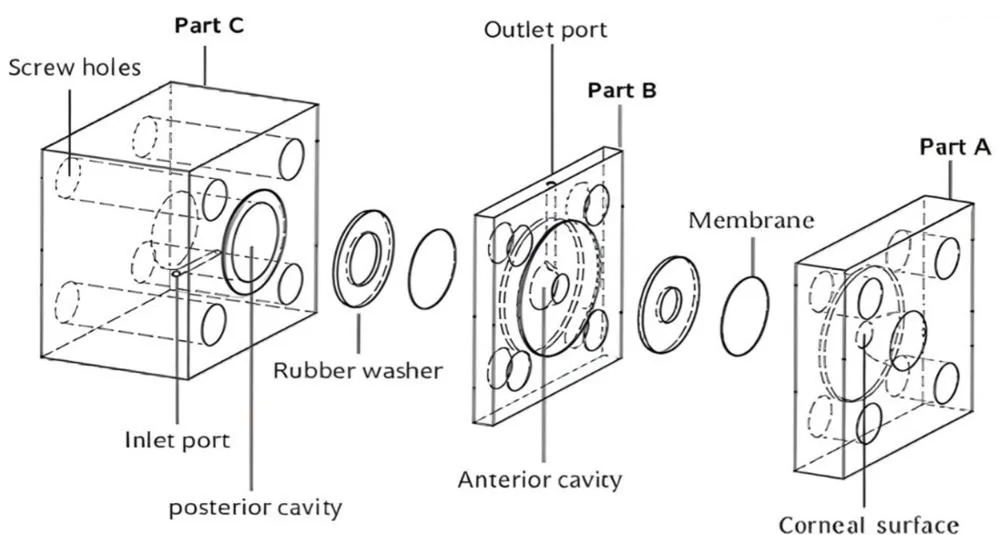
An exploded view of the *in vitro* eye-flow model

 The flow of the pumped balanced salt buffer is consistently directed from the posterior chamber to the anterior chamber, and the effluent from the device is collected in the third part of the setup. After 24 hours, the concentrations of the drug within the respective compartments were analyzed using HPLC, as previously described. Correct performance of the mentioned setup was tested with a methylene blue solution (1.2 mM) before running the test.

####  In Vivo Release Test

*In vivo* release testing was conducted on three NZW rabbits, each weighing between 3-5 kg, under controlled environmental conditions, which included a temperature of 25 °C, regulated ambient humidity, a standardized diet, and a 12-hour light-dark cycle over a period of 4 consecutive days. Two of the rabbits received 50 µl of the final ALA formulation, administered every 12 hours in both eyes, while the control rabbit received 50 µl of normal saline at the same intervals. Following the completion of the treatment regimen, the rabbits were euthanized on the fifth day, 12 hours after the last instillation, and samples of their aqueous humour and vitreous humour were subsequently collected by an ophthalmologist. These samples were analyzed for the presence of ALA using the HPLC method as described before.

####  Accelerated and Thermodynamic Stability Studies

 The thermodynamic stability test of synthesized nanoparticles is conducted through the implementation of three distinct phases: thermal cycling, centrifugation, and freeze-thaw cycles. The initial phase consists of six cycles, each cycle comprising 48 hours at 45 °C and 48 hours at 4 °C, respectively. The subsequent phase involves centrifugation for 30 minutes at a speed of 3500 rpm. The final phase includes three cycles, with each cycle consisting of two 48-hour intervals at -20 °C and 25 °C. For accelerated stability testing, samples produced by the microfluidic technique were placed under an inert atmosphere and stored for six months at two distinct temperatures of 4 °C and 45 °C. During this period, samples were collected at three time points: the initial time, the end of three months, and the end of six months. The samples were then analyzed for physical instability.^22,23^

####  Ocular Irritancy Studies

 A study on ocular irritancy was conducted involving eight New Zealand rabbits, each with a normal weight ranging from 3 to 5 kg. The experiment was carried out under controlled conditions, including a temperature of 25 °C, ambient humidity, a standard diet, and a 12-hour light-dark cycle over a duration of 10 days. All procedures conducted on rabbits and their conditions during the experiment adhered to the principles and guidelines established by the ethical research committee at Mashhad University of Medical Sciences (ethical code: IR.MUMS.PHARMACY.REC.1402.036). Prior to the initiation of the experiment, the formulations—both containing and devoid of ALA—were filtered and sterilized using a 0.45-micron filter. The rabbits were divided into two distinct groups. The first group comprised six rabbits, which received ALA-containing ophthalmic nanoemulsion into the left eyes and ALA-free cationic nanoemulsion in their right eyes. The second group consisted of two rabbits that received normal saline instillation in both eyes. Every 12 hours, 50 microliters of the sample were instilled into the eyes for 10 days. On the eleventh day, 12 hours after the final instillation, all eyes were examined by an ophthalmologist through a modified version of Draize technique by Morsi and her colleagues for signs of inflammation and reactions in the conjunctiva, cornea, iris, and eyelids.^22,24^ The ocular irritancy assessment was conducted by considering 0 (absence) to 3 (highest) grades for any clinical reaction (Table 2). The ocular irritation index was determined by aggregating the individual clinical scores. A score of 2 or 3 in any single category, or an overall score exceeding 4, was interpreted as indicative of clinically significant irritation. Subsequently, following ethical guidelines, the rabbits were euthanized using carbon dioxide gas. The ophthalmologist then selected two eyes from the six that received the ALA-containing formulation, two from the six that received the ALA-free formulation, and two from the four that received normal saline for pathologic evaluations. After being rinsed with a balanced salt solution, the samples were preserved in formaldehyde. Transverse sections of the samples were prepared by a qualified specialist, and these sections were subsequently evaluated under hematoxylin and eosin staining and light microscopy by a pathologist.

**Table 2:** The modified version of Draize grading scale for clinical evaluation of ocular irritation

**Clinical parameter**	**Observation**	**Score**
Conjunctival discharge	Normal	0
	Slight discharge	1
	Severe discharge covering a small area around the cornea	2
	Severe discharge covering a large area around the cornea	3
Conjunctival chemosis	Normal	0
	Slight chemosis including nictitating membrane	1
	Severe chemosis with the eye partially closed	2
	Severe chemosis with the eye closed	3
Conjunctival redness	Blood vessels normal	0
	Some blood vessels definitely hyperaemic	1
	Diffuse color, individual vessels not easily discernible	2
	Diffuse beefy red	3

## Results

###  Size and Zeta Potential

Table 3 presents the sizes corresponding to TFRs of 10, 12, 14, 16, and 18. Based on the findings, a constant ratio of 1:9 for the oil to aqueous phase, along with a temperature of 65 °C, yielded optimal results; therefore, these parameters were maintained. The results indicate that a TFR of 18 produced the most favorable size. Additionally, it is noteworthy that all outputs exhibited a positive zeta potential, which can be attributed to the inclusion of CTK as both a preservative and a cationizing agent.

**Table 3:** Size and zeta potential of ALA cationic nanoemulsions produced at different TFRs.

**Total Flow Rate (ml/min)**					
	**10**	**12**	**14**	**16**	**18**
Z-Average (nm)	208.7 ± 3.1	175.3 ± 0.6	175.2 ± 1.9	159.8 ± 2.7	166.9 ± 1.4
PDI	0.377 ± 0.074	0.112 ± 0.006	0.276 ± 0.030	0.318 ± 0.055	0.253 ± 0.024
Zeta potential (mV)	18.8 ± 2.2	16.5 ± 1.0	26.0 ± 1.5	11.3 ± 1.7	26.5 ± 1.3

###  FESEM Electron Microscopy

 The FESEM images (Figure 2) demonstrate that the nanoemulsion produced by the microfluidic technique contains particles with a spherical morphology. The observed particles are uniform in size, indicating size consistency and a low polydispersity index (PDI). The sizes recorded by FESEM further corroborate the results obtained from the DLS method using Malvern ZS Nanosizer.

**Figure 2:**
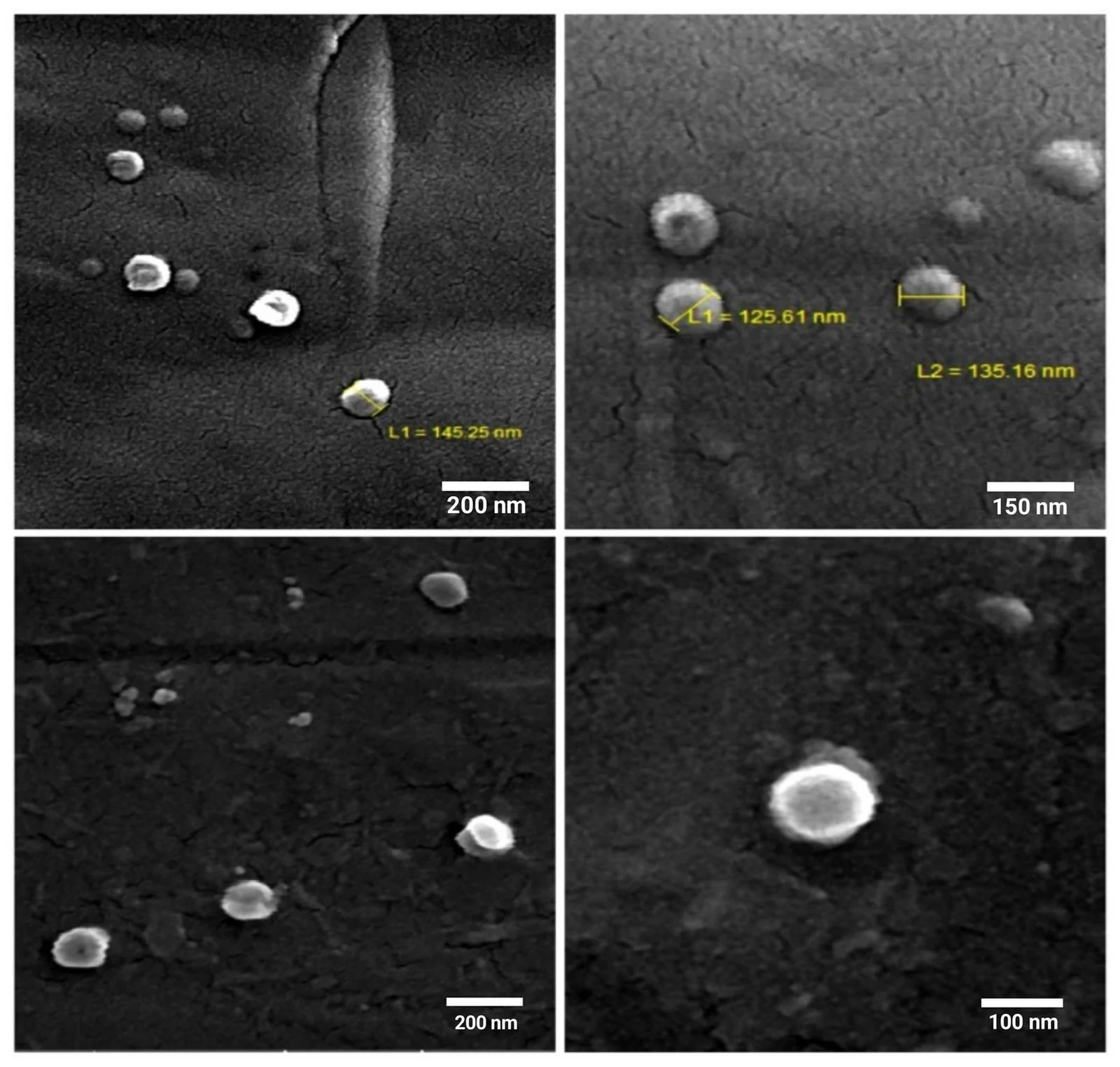
FESEM image of ALA cationic nanoemulsion

###  Determination of Incorporated ALA

 A standard curve (R^2^ = 0.9996) was established by utilizing HPLC and the previously described standards. The optimal ALA loading in the cationic nanoemulsion corresponds to TFR 18, which achieved a loading of 79.92% (7.9 mg/ml). According to specifications provided by Sigma, the solubility of ALA in water is reported to be 0.87 mg/ml.

###  Rheological Properties

 The rheological properties of the cationic nanoemulsion formulated at a TFR of 18 exhibited non-Newtonian pseudoplastic behavior, which is similar to human tears (Figure 3). Viscosity measurements of the investigated formulation, conducted at 34 ± 0.5°C across a shear rate range of 0–900 s⁻¹, are represented in Table 4.

**Figure 3:**
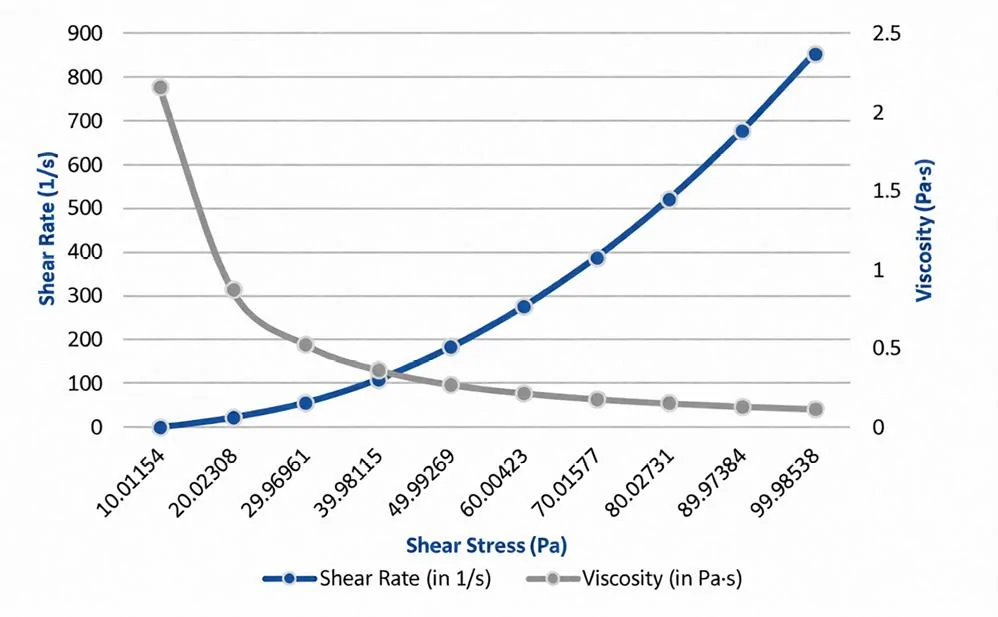
Rheogram of the cationic ALA nanoemulsion in TFR 18

**Table 4:** Rheometer data for ALA cationic nanoemulsion prepared in TFR18

**Time (s)**	**Shear Rate (1/s)**	**Shear Stress (Pa)**	**Viscosity (Pa·s)**
1	0	0	0
2	4.632108	10.01154	2.161335617
3	22.877811	20.02308	0.87521835
4	56.86855	29.96961	0.526997963
5	109.816333	39.98115	0.364072893
6	183.41237	49.99269	0.272569893
7	276.10617	60.00423	0.217323032
8	388.8492	70.01577	0.180058928
9	522.510303	80.02731	0.153159296
10	678.229432	89.97384	0.132659887
11	854.911819	99.98538	0.116954027

###  pH Measurements

 The final pH of ALA cationic nanoemulsion produced by the microfluidic device in TFR 18 was found to be in the acidic range of 4.5 to 5.0, which is attributable to the acidic properties of ALA. Consequently, the formulation was adjusted to a pH of 6.6 ± 0.1 using 0.1N M NaOH.

###  Refractive Index Measurements

 The cationic drug-loaded nanoemulsion, prepared at TFR 18, exhibited a refractive index of 1.34925 nd, which is equivalent to a Brix value of 10.9.

###  Osmolality Determination

 The final formulation generated by the microfluidic device in TFR 18 exhibits an osmolality of 583.84 ± 2.30 mOsm/kg, which is considered acceptable and does not induce irritation or discomfort in the eye.

###  Ex Vivo Release Test

 The release test of the formulation was conducted according to the previously described setup. The physiological intraocular clearance of the eye occurs from the posterior to the anterior segments. In contrast, the administered drug traverses in the reverse direction, specifically from the corneal surface to the posterior segments of the eye. In this context, a concentration of 1.2 mM methylene blue was employed to assess the efficacy of the described setup. As illustrated in Figure S3, after a duration of 24 hours, both the outlet and posterior compartments of the *in vitro* release setup exhibited a blue coloration, confirming the proper operation of the system as outlined. After ensuring that the system was functioning correctly, the formulation prepared in TFR 18 was utilized for the release test. A volume of 500 µl of the formulation was applied to the corneal surface. After 24 hours, 21.04% of the applied ALA was detected in the third part of the setup. In contrast, 38.17% of the ALA was detected in the second part of the setup, component C. Overall, 59.3% of the ALA was detected in the compartments and successfully passed through the cornea.

###  In Vivo Release Test

 After four days of instillation (50 microliters of sample every 12 hours in both eyes), on the fifth day, the ophthalmologist obtained samples of the aqueous humor and vitreous humor. The samples were diluted 1:1 with deionized water and analyzed using HPLC following centrifugation for five minutes at 13,000 rpm. The mean concentration of ALA found in the aqueous humor of rabbits was 54.19 µg/ml, while the mean concentration of ALA in the vitreous humor was 9.75 µg/ml.

###  Accelerated Thermodynamic Stability Studies

 The thermodynamic stability of the formulations was evaluated using the previously described methodology. After 6 cycles of heating and cooling, along with 30 minutes of centrifugation and subsequent freeze-thaw cycles, the formulations exhibited slight creaming and lost their uniformity. A z-average of 119.3 nm and a PDI of 0.652 were reported along with a zeta potential of +33.3 mV after three thermodynamic stability cycles.

 To assess accelerated stability, the formulations were stored at temperatures of 45 °C and 4 °C. The thermodynamic state of the samples stored at 45 °C was replicated, resulting in creaming due to the elevated temperature, and the formulations lost their uniformity, similar to the behavior observed in thermodynamic formulations. In contrast, the samples maintained at refrigeration temperature (4 °C) showed no signs of cracking, creaming, or color alteration, thereby preserving their uniformity. No phase separation was observed, and the nanoemulsion system remained stable at the refrigerated temperature. Also, DLS analyses conducted at the end of the third month revealed a Z-average of 168.5 nm, a PDI of 0.059, and a zeta potential of 21.1 mV. By the end of the six months, the z-average was measured at 166.7 nm, with a PDI of 0.048 and a zeta potential of 21.0 mV (Table 5). These results indicate that the formulations stored under refrigeration demonstrated satisfactory stability and successfully met the criteria for stability assessment.

**Table 5:** Size, polydispersion index, and zeta potential of formulations at TFR 18

**Time**	**Z-Average (nm)**	**PDI**	**Zeta Potential (mV)**
Zero time	166.9 ± 1.4	0.253 ± 0.002	26.5 ± 1.3
After 3 months	168.5 ± 0.9	0.059 ± 0.001	21.1 ± 1.1
After 6 months	166.7 ± 0.7	0.048 ± 0.002	21.0 ± 1.0

###  Ocular Irritancy Studies

 Before the initiation of the experiment, all rabbits underwent a comprehensive evaluation conducted by an ophthalmologist, which revealed no signs of inflammation or damage in the eyelids, cornea, iris, conjunctiva, or anterior chamber of the eyes. On the eleventh day, 12 hours after the final instillation, a re-evaluation of the eyes was performed by the ophthalmologist, and the absence of inflammation and redness following the instillation of the formulation was reported. The observation indicates that the formulation was well tolerated by the ocular tissues of the rabbits. Following the ophthalmological assessment, corneal cross-sections were prepared and stained with Hematoxylin and Eosin for examination under a light microscope. The analysis included two eyes that received the formulation containing ALA, two eyes that received the formulation without ALA, and two eyes that received normal saline (Figure 4). The histological examination of the corneal tissue revealed a healthy epithelial layer characterized by stratified squamous cells. Additionally, the collagen fibers within the stroma exhibited a uniform orthogonal arrangement, accompanied by spindle-shaped keratinocytes.

**Figure 4:**
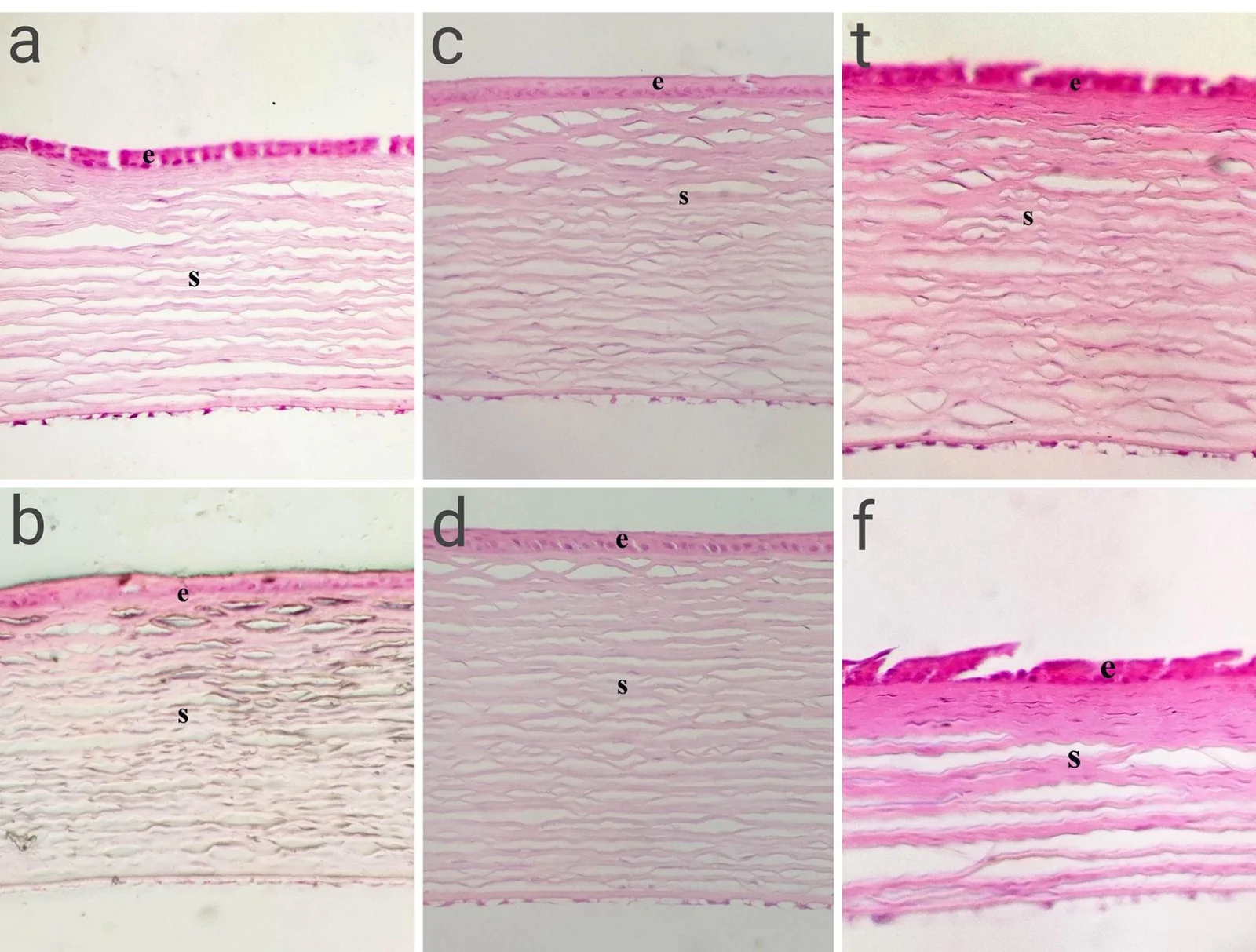
Light microscopic images of corneal cross-sections illustrate the epithelial layer (e), which consists of stratified squamous cells, as well as the collagen fibers present in the stroma (s). The images depict eyes that were administered ALA-containing cationic nanoparticles (a, b), eyes that received ALA-free cationic nanoparticles (c, d), and eyes treated with normal saline (t, f). (Hematoxylin and Eosin × 400)

## Discussion

 One of the major challenges in ocular drug delivery is achieving effective penetration to the internal structures of the eye, particularly the posterior segments such as the vitreous humor and retina.^25^ The vitreous is a transparent gel filling the space between the lens and retina that plays a critical role in maintaining the eye’s spherical shape.^26^ Drugs to treat diseases like diabetic retinopathy or age-related macular degeneration need to overcome the vitreous humor, which is a substantial barrier.^25,27^ Nanoemulsions hold great potential as a drug delivery system for targeting the posterior segment of the eye because they can diffuse freely in biological tissues and facilitate drug permeation.^28^

 In the present work, a nanoemulsion of ALA was developed by a microfluidic technique, with desirable physicochemical characteristics, such as optimal particle size, high encapsulation efficiency, and controlled release patterns, in order to deliver ALA into the posterior segment of the eye as a localized antioxidant. The microfluidic approach proved to be an efficient and sophisticated method for nanoparticle production with precise and uniform size distributions, overcoming the limitations of conventional techniques, such as physical instability and variability in particle sizes.^29^ The product developed was found to possess the ability to release ALA in the posterior segment of the eye, as was evidenced through HPLC analysis for samples obtained from the vitreous and aqueous humor of rabbits that indicated the presence of alpha-lipoic acid in these ocular compartments.

 In the first step, approximately 40 initial formulations were prepared based on data from the *Handbook of Pharmaceutical Excipients,*^30^ and another related study by Mahmoudi et al.^18^ These preliminary formulations were then subjected to achieve the final optimized formulation, which exhibited enhanced stability and activity. The incorporation of minimal ethanol to solubilize ALA, non-ionic surfactants (Span 80 and Tween 80), vitamin E as an antioxidant, cetalkonium chloride as a cationic agent and preservative, and glycerin to enhance viscosity and tonicity collectively contributed to the formulation’s efficacy and stability.

 The nanoemulsion, prepared at TFR 18, had a particle size of 166.9 ± 1.43 nm, which went up to 208.7 ± 3.11 nm at reduced flow rates, showing an inverse relationship between flow rate and particle size. Also, smaller particle sizes generally correlate with improved tissue penetration, as long as stability is ensured.^31^ The formulation’s zeta potential of +33.3 mV at the optimal flow rate ensured colloidal stability by preventing particle aggregation through electrostatic repulsion.

 This positive charge also facilitated electrostatic interactions with the negatively charged corneal surface, thereby enhancing residence time and bioavailability.^32^ Research shows that the typical refractive index range for the human cornea, including the overlying tear film and lens, is approximately 1.33 to 1.44 nd.^33^ Conversely, a refractive index exceeding 1.476 nd is not advisable for ophthalmic solutions.^34^ The refractive index of our formulated product (1.34925 nd, equivalent to 10.9 Brix) was within the acceptable range for ocular formulations (1.33-1.44). Therefore, it is compatible with corneal tissue and minimizes visual disturbances.

 Our formulation displayed non-Newtonian pseudoplastic behavior, similar to human tear fluid, with a decrease in viscosity upon application of increasing shear stress. This shear-thinning property is ideal for ophthalmic formulations, as it reduces viscosity during instillation, facilitating ease of administration, and at the same time, increases viscosity upon exposure to the ocular surface, prolonging residence time. Viscosity measurements performed at 34 ± 0.5 °C, approximating the corneal surface temperature of rabbits (32-34 °C), confirmed the suitability of the formulation for ocular use. The physiological osmolarity of human tears is established at 295 ± 10 mOsm/kg. Consequently, eye drops are classified into three categories: iso-osmolar, hyper-osmolar, and hypo-osmolar, based on their osmolarity range.^35^ In contrast, non-isotonic ophthalmic solutions with osmolality levels below 100 mOsm/kg or above 650 mOsm/kg may irritate.^36^ Thus, the eye drops produced must adhere to the specified osmolality range to minimize ocular irritation. The osmolarity of 583.84 ± 2.30 mOsm/kg fell in the desired range (100–650 mOsm/kg) of ophthalmic preparations and hence would cause minimal irritation. The degree of ocular irritation caused by eye drops is influenced by several factors, including the pH of the drops, the volume administered, the duration of contact with the cornea, and the buffering capacity of the formulation. Ideally, the pH of eye drops should be maintained within the range of 6.5 to 7.8, with a pH of 7.4 ± 0.1 being the most favorable, as it closely resembles the pH of natural human tears.^37,38^ However, it is essential to consider the stability of the ingredients and the base pH of the formulation when determining the final pH. The pH of our formulation was initially in the acidic range (4.5-5.0) due to the acidic nature of ALA, but was subsequently adjusted to 6.6 ± 0.1 by the addition of 0.1 N NaOH, in adherence to the optimum range (6.5-7.8) necessitated for ocular comfort and stability and due to the formulation’s limited buffering capacity, it is readily buffered within the ocular environment after instillation, thereby minimizing irritation and ensuring acceptability.^39^ Thermodynamic stability tests indicated that the nanoemulsion was stable when stored cold (4 °C), without any phase separation, color change, or physical instability. However, at high temperatures, the preparation exhibited particle aggregation and loss in uniformity, emphasizing the requirement of cold storage for maintaining stability. Laser light scattering measurements supported these observations, where minimal particle size and polydispersity changes occurred at 4 °C. FESEM imaging also verified the spherical shape and uniform size distribution of the nanoparticles, which was consistent with laser light scattering analysis and further validated the accuracy of the microfluidic technique.

 A previous research by Mahmoudi et al.^18^, cationic nanoemulsion containing ALA was prepared using a conventional method, and the physicochemical properties of the prepared formulation were evaluated. They reported an ALA-nanoformulation with 10% drug release & a size of 289.14 nm. In comparison to our study, the nanoemulsion particle size prepared by microfluidics was smaller (166.9 nm vs. 289.14 nm), and drug release was higher (59.3% vs. 10%). Furthermore, the encapsulation efficiency of ALA in the nanoemulsion was 79.92 ± 0.5%, which is much higher than that of traditional methods (61%) reported. This high encapsulation, coupled with a 9-fold increase in ALA’s aqueous solubility (from 0.87 mg/mL), highlights the formulation’s potential for effective ocular drug delivery. In another study by Garner et al.^40^, they prepared a herbal formulation called DIOPTIN® based on 18-lipoic acid choline ester. The purpose of this formulation was to increase the water solubility and the passage of lipoic acid through the cornea. The safety of the DIOPTIN® formulation was investigated in a 90-day animal trial on New Zealand White rabbits. The tolerability and non-irritability of the formulation were confirmed during this trial. We also conducted a safety experiment with our ALA-cationic nanoemulsion, using a design similar to that of DIOPTIN® drops, on New Zealand rabbits. The tolerability and non-irritability of our cationic nanoemulsion formulation were also confirmed during this study. Ocular irritation tests in rabbits did not show any inflammation or erythema, while histological examination of corneal sections demonstrated the integrity of the epithelial layer and normal arrangement of collagen fibers in the stroma. These findings indicate that the ALA-loaded nanoemulsion is biocompatible and safe for ocular administration. Additionally, the formulation’s pH, osmolarity, and viscosity were maintained within a range that minimized irritation while ensuring bioavailability, which is comparable to the properties of human tear fluid.

 The release test of DIOPTIN® was conducted on New Zealand rabbits for 90 minutes. During this test, the net penetration of the drug into the lens was recorded as 0.28%. The absorption constant (Ki) was 0.15 per minute, and the release constant (Ke) was 0.07 per minute for DIOPTIN®.^40^ Our in vitro corneal permeation test demonstrated a drug release of 59.3 ± 0.5%, a substantial improvement over the 0.28% reported for DIOPTIN®. In addition, our in vivo studies performed on New Zealand white rabbits also confirmed the efficacy of the formulation, with ALA concentrations of 54.19 µg/ml in the aqueous humor and 9.75 µg/ml in the vitreous humor, hence proving effective delivery of our formulation to the posterior segments of the eye. These results, supported by HPLC analysis, suggest that the nanoemulsion’s small particle size, low polydispersity, and cationic charge enhance corneal penetration and intraocular bioavailability, which can effectively overcome the obstacles relating to drug delivery to the vitreous body and retina. Cagini et al.^8^ also conducted a study to determine the concentration of ALA in human aqueous humor after topical application of its eye drops. They performed their research on patients who were candidates for cataract surgery. Their results indicated that ALA is naturally present in the aqueous humor of the eye, and after instilling eye drops containing it in the eye, only the amount of this substance in the eye increases. Their peak concentration of ALA was found to be 5.2 ± 2.9 ng/ml in human aqueous humor. However, our formulation discussed here achieved high concentrations of 54.1 µg/ml, highlighting its potential for enhanced therapeutic efficacy with statistically significant differences (*P* < 0.0001).

 These benefits are associated with the microfluidic technique, which can produce uniform nanoparticles with higher stability.^41^ To our knowledge, this paper presents the first research survey on the application of microfluidic techniques to produce ALA-nanoemulsions for ocular drug delivery. Although we have several limitations. Our study did not evaluate the impact of some environmental factors, such as light exposure, tear dynamics, blink rate, and ocular surface temperature, on drug delivery. We suggest future studies incorporate these parameters to simulate physiological conditions better. Also, long-term safety studies need to be conducted to analyze the possible side effects associated with chronic use of nanoemulsions. Besides, investigating the therapeutic efficacy of the ALA-nanoemulsion in animal models of ocular diseases, such as diabetic retinopathy or age-related macular degeneration, would further validate its clinical potential. Additionally, exploring formulations stable at room temperature could enhance practical applicability.

## Conclusion

 In this research, we discovered that the preparation of the cationic nanoemulsion via microfluidics resulted in an approximate nine folds enhancement in the solubility of ALA in aqueous solutions, successfully achieving the desired particle size, entrapment efficiency, and other relevant characteristics. Accelerated stability test demonstrated that the formulation remained stable under refrigerated conditions. Furthermore, *in vivo* experiments conducted on rabbits confirmed the formulation’s compatibility and the absence of ocular irritation. Both *in vivo* studies on rabbits and ex vivo investigations on bovine corneas revealed an effective release profile of ALA, as well as a considerable enhancement in drug delivery to the posterior segments of the eye, including the vitreous body. Overall, the physicochemical properties of the developed nanoemulsion, along with the findings from *in vivo* and *ex vivo* studies, suggest that the final formulation is both safe and stable under the described conditions. These favorable attributes were achieved through the microfluidic technique, which involved fewer manufacturing steps compared to conventional methods. The formulation, characterized by sustained drug release and improved interaction and penetration at the ocular surface, presents a promising avenue for further research and potential commercialization.

## Competing Interests

 The authors have no relevant financial or non-financial interests to disclose.

## Consent For Publication

 The authors affirm that no human research participants were used and thus informed consent for publication is not required. All authors concur with the publication of the manuscript.

## Data Availability Statement

 The datasets generated during the current study are available from the corresponding author on reasonable request.

## Ethical Approval

 The entire study was conducted following the guidelines provided by the Institutional Research Ethics Committee of the School of Pharmacy at Mashhad University of Medical Sciences, under approval number IR.MUMS.PHARMACY.REC.1402.036.

## Supplementary File

Supplementary file contains Figures S1-S3.
