# Supplementary material for: ALA Nanoemulsion Prepared By Microfluidic for Ocular Delivery: In-Vitro and In-Vivo Studies
Source: Adv Pharm Bull. 2026 May 9;16(3):606–16. doi: 10.34172/apb.46273 (PMC13745752; doi:10.34172/apb.46273)
Supplement: Supplementary file 1 [file apb-16-606-s001.pdf]

Adv Pharm Bull 2026;16(3):606-S1
doi:10.34172/apb.46273

https://apb.tbzmed.ac.ir

Supplementary data

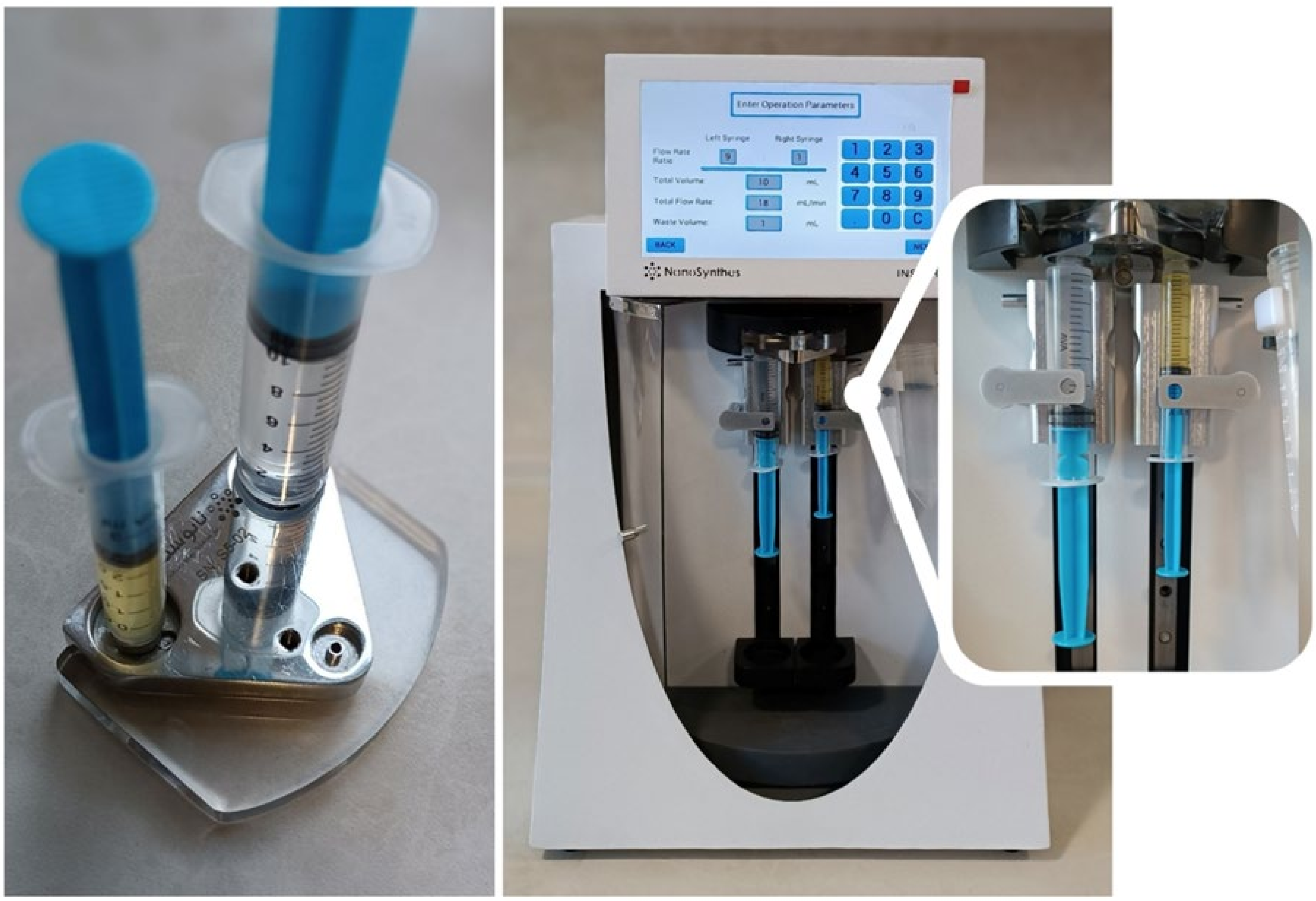

Figure S1. The INSIGHT microfluidic apparatus, along with the micromixing cartridge that features two interconnected syringes, one filled with aqueous phase and the other with oil phase.

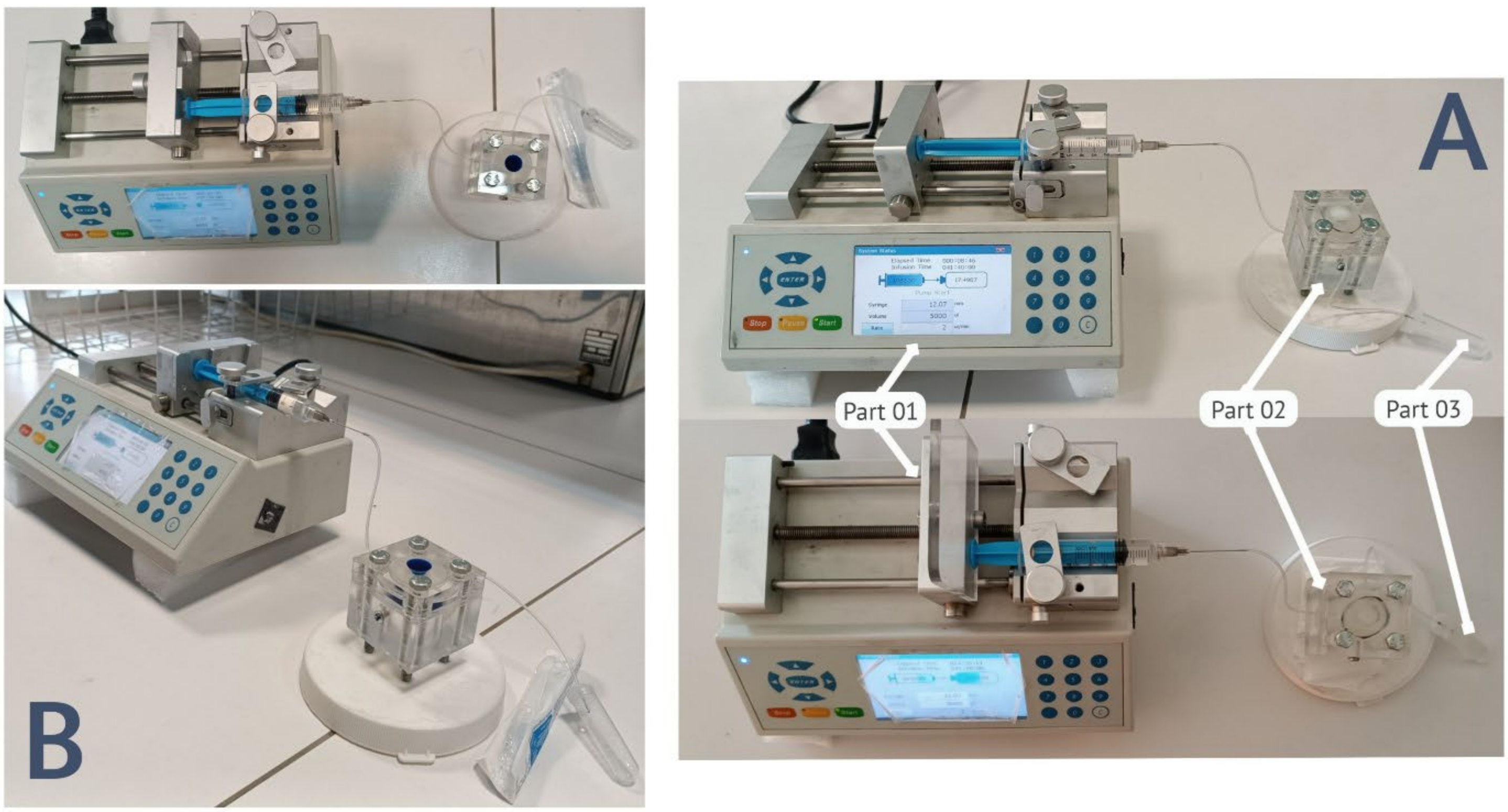

Figure S2. The experimental configuration used for the release test of the ALA cationic nanoemulsion consists of three components: Part 1 involves a pump; Part 2 includes an apparatus designed to simulate the various compartments of the eye; and Part 3 pertains to the output of a buffered solution that is pumped from the aforementioned device, B: ex vivo release experiment conducted utilizing 500 microliters of a 1.2 mM methylene blue solution

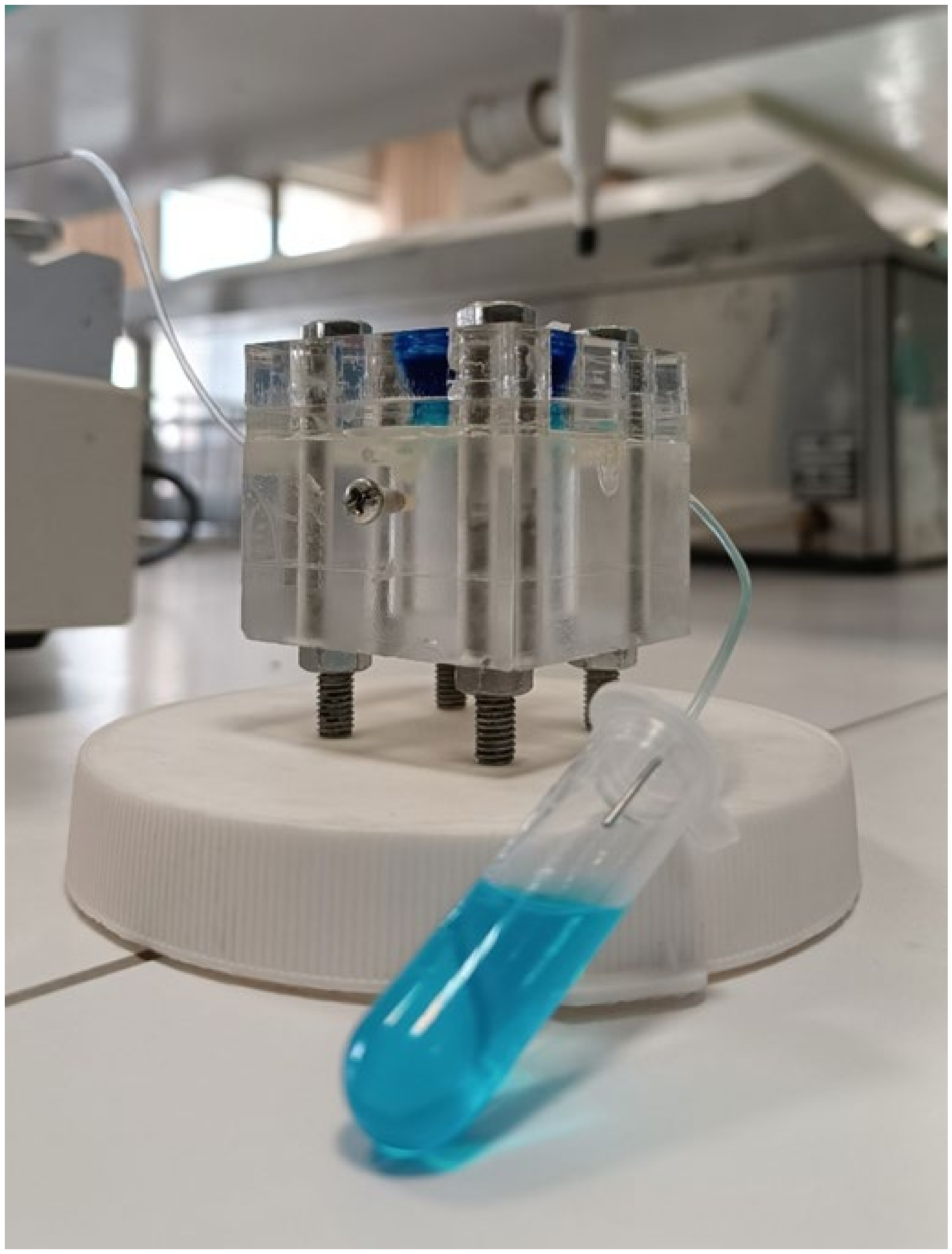

Figure S3. An image of the release system after 24 hours of operation, featuring a concentration of 1.2 mM methylene blue and a flow rate of 2 μL/min of balanced salt buffer, along with the color changes observed in various components of the system.
